# Morphological and Behavioral Abnormalities Induced by Hydrogen Peroxide in *Drosophila melanogaster*

**DOI:** 10.3390/biology14091122

**Published:** 2025-08-25

**Authors:** María Llasbeth Hernández-Calderón, Alondra Gallegos-Moreno, Aneet Yamely Miranda-Camacho, Claudia Linette Sánchez-Jiménez, Sandra Díaz-Barriga-Arceo, Jorge Alejandro Aguirre-Joya, Cristian Torres-León, David Ramiro Aguillón-Gutiérrez

**Affiliations:** 1 Facultad de Estudios Superiores Cuautitlán, Laboratorio de Citogenética Humana, Universidad Nacional Autónoma de México, Av. 1o de Mayo S/N, Santa María las Torres, Cuautitlán Izcalli 54740, Mexico; llasbeth.hdez@cuautitlan.unam.mx (M.L.H.-C.); 318264457@cuautitlan.unam.mx (A.G.-M.); aneetyamely1234@gmail.com (A.Y.M.-C.); 317069242@cuautitlan.unam.mx (C.L.S.-J.); sadibar@unam.mx (S.D.-B.-A.); 2 Centro de Estudios e Investigaciones Interdisciplinarias, Universidad Autónoma de Coahuila, Centro Cultural 2° piso, Ciudad Universitaria, Carretera México km. 13, Arteaga 25350, Mexico; 3 Facultad de Estudios Superiores Cuautitlán, Laboratorio de Genética y Toxicología, Universidad Nacional Autónoma de México, Carretera Cuautitlán-Teoloyucan Km. 2.5, Cuautitlán Izcalli 54714, Mexico; 4 Centro de Investigación y Jardín Etnobiológico, Universidad Autónoma de Coahuila, Dr. Francisco González # 37, Viesca 27480, Mexico; jorge_aguirre@uadec.edu.mx (J.A.A.-J.); ctorresleon@uadec.edu.mx (C.T.-L.)

**Keywords:** LC_50_, teratogenesis, oxidative stress, behavioral assays, teratogenic index

## Abstract

Congenital developmental defects are among the postnatal consequences of early exposure to hydrogen peroxide or other teratogens that induce oxidative stress, highlighting a potential mechanistic link between oxidative stress, redox signaling, and developmental processes. This study evaluated the morphological and behavioral abnormalities induced by hydrogen peroxide in the *Drosophila melanogaster* model, as well as its teratogenic index. The results demonstrated that hydrogen peroxide induces morphological abnormalities in adult wings, legs, and abdomen, as well as necrosis and developmental disruptions during larval and pupal stages. A median lethal concentration (LC_50_) of 0.16% and a teratogenic index (TI) of 0.44 were calculated when considering anomalies at any development stage; a TI of 0.21 was obtained when considering only adult abnormalities. Regarding behavioral changes, an increase in locomotor activity was observed in both larvae and adults, with significantly greater activity recorded in adult females than in males. These findings suggest that hydrogen peroxide can induce both morphological and behavioral abnormalities in *D. melanogaster*, although it presents a low teratogenic index.

## Simple Summary

Numerous environmental pollutants and substances encountered in our daily lives can induce oxidative stress, a physiological imbalance characterized by an overproduction of free radicals relative to antioxidant defenses. This imbalance can lead to detrimental effects on biological systems, including damage to essential biomolecules such as DNA and proteins, as well as developmental anomalies. Therefore, it is crucial to investigate the morphological impact and behavioral consequences of oxidative stress using established biological models. The fruit fly (*Drosophila melanogaster*) is a useful biological model for investigating the mechanisms of oxidative stress and its biological consequences.

## 1. Introduction

Currently, various emerging pollutants, such as micro- and nanoplastics [1,2,3,4,5,6], their associated additives [7], and heavy metals [8], can individually or collectively cause harm to human health and other organisms by inducing oxidative stress. An imbalance between the excessive production of free radicals and the diminished activity of antioxidant defense systems characterizes this condition. Free radicals are atoms or molecules that contain unpaired electrons and are generated as byproducts of normal cellular metabolism or in response to environmental stressors. These unstable species can react with other molecules by accepting or donating electrons [9].

Hydrogen peroxide (H_2_O_2_) is a reactive oxygen species (ROS) commonly employed as an oxidizing agent in the medical and chemical industries. However, it is also endogenously synthesized by diverse organisms, where it functions as a signaling molecule involved in regulating various physical and biochemical processes [10,11,12]. The oxidative effects of H_2_O_2_ are primarily mediated through its conversion into highly reactive hydroxyl radicals [13]. These radicals can selectively oxidize redox-sensitive amino acid residues in proteins, thereby modulating key signaling pathways. Additionally, excessive accumulation of H_2_O_2_ may disrupt cellular redox homeostasis, leading to nonspecific and irreversible damage to biomacromolecules such as nucleic acids, proteins, and lipids [14]. This oxidative imbalance has been strongly associated with cellular senescence, aging processes, and reduced organismal lifespan [15].

Early-life exposure to H_2_O_2_ can result in postnatal consequences such as congenital malformation (teratogenesis), functional impairments, and disease [16]. Among known teratogens, many act by generating oxidative stress, suggesting an important mechanistic link between oxidative stress, redox signaling, and development [17,18]. Several xenobiotics have been shown to induce teratogenic effects via free radical-mediated mechanisms in animal models. Notable examples include benzo[a]pyrene, anticonvulsant drugs, thalidomide, methamphetamine, Adriamycin, 7-hydroxy-2-acetylaminofluorene, and paracetamol, all of which have been associated with oxidative stress-induced embryotoxicity [19].

To characterize the teratogenic potential of a xenobiotic, its teratogenic index (TI) can be calculated, defined as the ratio between the lethal concentration 50 (LC_50_) and the effective concentration 50 (EC_50_) [20]. To date, no standardized comparative scale has been reported that allows extrapolation of teratogenic index results from insects to mammals or other vertebrates. However, Lee et al. (2013) [21] made a preliminary attempt at categorization using the zebrafish model, establishing that a TI < 1.4 corresponds to Food and Drug Administration (FDA) category C, indicating that the xenobiotic under study may cause possible adverse effects on the fetus, whereas a TI > 2 corresponds to FDA category D, which indicates evidence of fetal risk [22].

The current approach to teratogenesis studies, known as environmental teratogenesis, considers both morphological abnormalities and behavioral alterations. In insects such as *Drosophila melanogaster*, which display a range of complex behaviors, larval locomotor activity (referred to as foraging behavior) and adult flight behavior may be evaluated. These behavioral patterns are regulated by circadian rhythms, which in turn are modulated by redox-active compounds [23]. The present study aims to explore the link between oxidative stress induced by various xenobiotics, such as environmental contaminants, and developmental defects, as well as behavioral effects. Hydrogen peroxide is used as an experimental substance, to extrapolate the results to contaminants that act through similar mechanisms of action.

## 2. Materials and Methods

### 2.1. Biological Material

All experiments were conducted using wild-type *Drosophila melanogaster* (^+/+^) strains obtained from the Fly Bank at the Faculty of Sciences of the Universidad Nacional Autónoma de México (UNAM, Mexico City, Mexico). These strains had been adapted over several generations to the conditions of the Human Cytogenetics laboratory at the Facultad de Estudios Superiores Cuautitlán.

### 2.2. Obtaining Third-Instar Larvae of Drosophila Melanogaster

Crosses of 30 males and 30 females were established in glass flasks containing 25 mL of yeast-based culture medium (composed of 8.25 g yeast, 8.75 g sucrose, 13.12 g corn flour, 1.25 g carrageenan, 0.62 g gelatin, 0.5 mL of 12% nipagin, 0.5 mL of propionic acid, and 156 mL of distilled H_2_O). After 12 h, adult flies were removed to allow larval development. Breeding was conducted under controlled laboratory conditions at 25 ± 2 °C, 35–40% relative humidity, and 12/12 h light/dark photoperiod.

### 2.3. Larval–Adult Viability and LC_50_ of H_2_O_2_

For H_2_O_2_ exposure, a culture medium based on dehydrated potato flakes was hydrated with the corresponding experimental concentrations (0.03, 0.125, 0.25, 0.5, 0.75, 1, 1.25, 1.75, 2.25, 2.5, 2.75, and 3%). Injectable water was used as a negative control. Each condition was performed in triplicate, with 50 third-instar larvae previously washed in PBS (pH = 7.4) per replicate. After seven days, emerged adults were collected, and larval-to-adult viability was assessed. The LC_50_ value was estimated using Probit regression analysis based on graphical methods.

### 2.4. Morphological Evaluation

Morphological assessments were conducted following the same exposure procedure described above, using H_2_O_2_ concentrations of 0.03, 0.125, 0.25, 0.5, 0.75, 1, and 1.25%. After adult emergence, flies were anesthetized with anhydrous ethyl ether and examined under a stereoscopic microscope (VELAB STEREO VE-S6, Velab Co., Pharr, TX, USA) to identify morphological abnormalities. Non-viable larvae and pupae were also collected and analyzed for anomalies at concentration of 1.25-3 %. Photographic documentation was carried out using a VELAB VE-LX1800 (VELAB Co., Pharr, TX, USA) camera and ToupView software (TOUPTEK PHOTONICS, version x64, 4.11.21973.20230107, ToupTek, Hangzhou, China). 

The effective concentration 50 (EC_50_) and teratogenic index were calculated under two scenarios: (1) the percentage of anomalies observed in live adults and (2) the percentage of anomalies across all developmental stages analyzed. The EC_50_ was calculated using a graphical method by constructing a concentration–response curve and extrapolating the concentration at which H_2_O_2_ produces 50% of the effect, namely, the induction of abnormalities. The teratogenic index (TI) was calculated using the formula reported by Tyl (2014) [20]: TI = LC_50_/EC_50_.

### 2.5. Behavioral Tests

To evaluate locomotor activity in third-instar larvae, the protocol described by Mishra & Barik. (2018) [24] was generally followed, with some modifications: actively feeding third-instar larvae that were exposed for four hours to culture medium containing 0.25% H_2_O_2_, then collected and rinsed with PBS (pH = 7.4).

After exposure, each larva was rinsed with PBS (pH = 7.4) to remove excess culture medium. Then, an individual larva was placed at the center of a Petri dish containing 1% agarose. Its movement was recorded for one minute. The distance traveled (cm) was quantified using ImageJ software (version 1.54d, National Institutes of Health, USA) [25]. Images were previously calibrated with a known scale, and measurements were performed using the line tool. The mean distance (cm) traveled by 20 larvae is reported.

For the adult negative geotaxis assay, the methodology described by Droso4 LATAM [26] was followed, as described below. Flies previously exposed to H_2_O_2_ at the third-instar larvae and allowed to mature for one week in an H_2_O_2_-free medium were anesthetized with ethyl ether and placed in a vertical glass column (245 mm x 32 mm). After a 15 min recovery period, the flies were gently tapped to the bottom of the column and allowed to ascend freely. A photograph was taken after 15 s, and the maximum height reached by each fly was recorded using the Manchester University scale by Prokop & Patel (2018) [27]. A total of 50 flies were analyzed in both the negative control and exposed groups.

### 2.6. Statistical Analysis

Statistical analysis was performed using SPSS version 29.0.2.0 [28] and Microsoft Excel 365. Data are reported as mean ± standard error (SE). For variables that did not follow a normal distribution, the Kruskal–Wallis test was used, followed by Dunn’s post hoc test with Bonferroni correction. Student’s *t*-test was applied for the larval motility behavioral assay, and the adult behavioral assay was based on the framework proposed by Prokop & Patel (2018) [27]. Statistical significance was set at *p* < 0.05.

## 3. Results

### 3.1. Larval–Adult Viability

**Figure 1:**
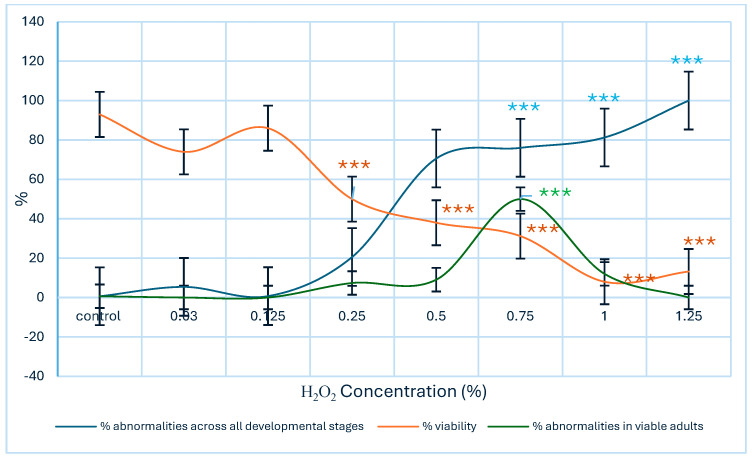
Comparison of larval–adult viability and the incidence of morphological abnormalities induced by H_2_O_2_ in a wild *Drosophila melanogaster* strain. Data are expressed as mean ± SE, with sample size (*n*) varying according to the viability percentage observed in each experimental group. Statistically significant differences were detected relative to the negative control (Kruskal–Wallis test *** *p* < 0.001).

A significant decrease in larval–adult viability (*p* < 0.001) was observed beginning at an H_2_O_2_ concentration of 0.25%. Viability continued to decline as the percentage of morphological anomalies in larvae, pupae, and adults increased, with statistically significant differences observed at concentrations of 0.75% or higher (*p* < 0.001). When considering only the frequency of anomalies in living adults, significant differences were also observed (*p* < 0.001). However, at higher concentrations, viability was markedly reduced, limiting the number of adults available for evaluation (Figure 1). Given that larval selection for viability assessment was performed randomly, the potential influence of sex on mortality could not be evaluated.

### 3.2. Morphological Evaluation

**Figure 2:**
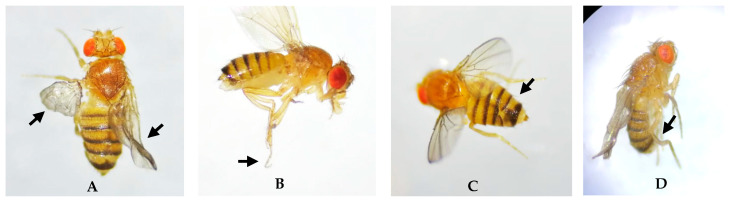
Morphological abnormalities induced by H_2_O_2_ in adult female wild *D. melanogaster*. (**A**) Wing abnormalities at 0.25% H_2_O_2_. (**B**) Right hind limb abnormality at 0.5% H_2_O_2_. (**C**) Abnormality in abdominal segment development. (**D**) Right hind limb abnormality at 0.75% H_2_O_2_. Images observed at 2× magnification. Arrows indicate the anatomical location of the fly exhibiting the morphological abnormality.

**Figure 3:**
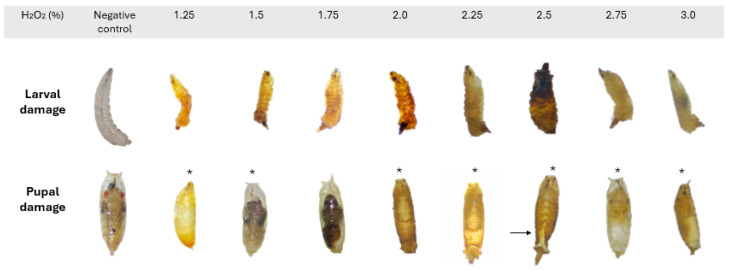
H_2_O_2_-induced abnormalities in third-instar larvae and pupae of wild *D. melanogaster*. * Abnormalities in respiratory spiracles. → Abnormal pupal case closure. Developmental defects became evident at H_2_O_2_ concentrations of 1.25% or higher. Images observed at 2× magnification.

The primary abnormalities observed in adults affected the wings, legs, and abdomen (Figure 2) and did not appear to affect either sex preferentially. In larvae, varying degrees of necrosis were recorded. In pupae, malformations included alterations in spiracle shape and length, absence of spiracle eversion, defects in pupal case closure, and atypical development of organisms (Figure 3). Due to the extent of damage observed at this stage, it was not possible to determine whether these abnormalities occurred more frequently in one sex over the other.

A statistical summary of the percentage of morphological abnormalities induced by hydrogen peroxide in *Drosophila melanogaster* is available in the Supplementary Materials (Table S1).

### 3.3. LC_50_ of H_2_O_2_

**Figure 4:**
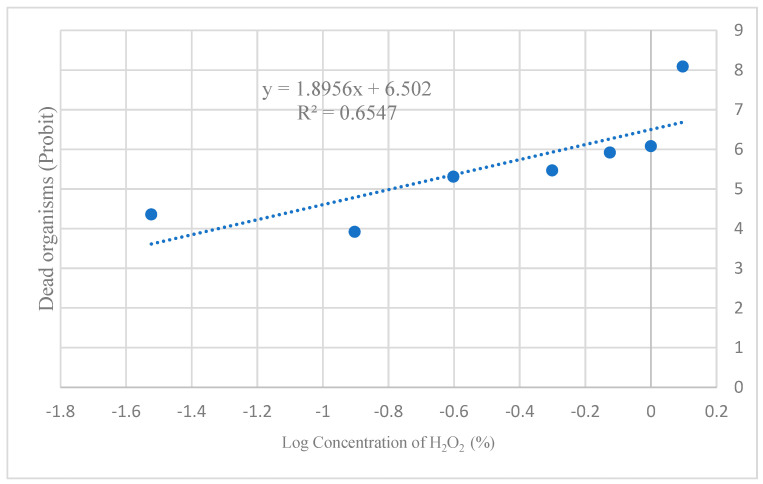
Determination of the LC_50_ for H_2_O_2_ in the *Drosophila melanogaster* model. The coefficient of determination (R^2^) indicates that 65% of the variability in organism mortality is explained by the H_2_O_2_ concentration to which they were exposed. This suggests that the model exhibits moderate predictive power and that additional variables are influencing the observed response.

Based on the concentration–mortality relationship, the LC_50_ for H_2_O_2_ was calculated as 0.16% (Figure 4).

### 3.4. Behavioral Assays

**Figure 5:**
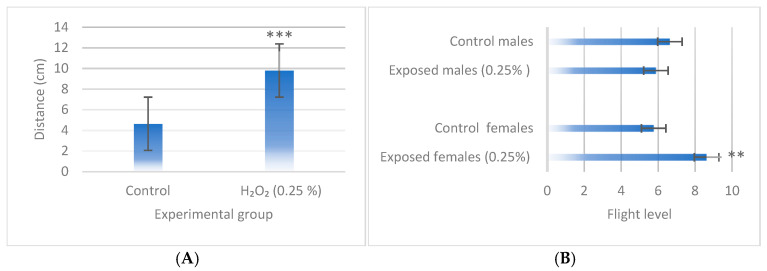
Behavioral tests in (**A**) larvae and (**B**) adults of wild-type *D. melanogaster* exposed to 0.25% H_2_O_2_. Data are presented as mean ± SE (*n* = 20 for larval behavior assay and *n* = 50 for adult behavior assays). Statistically significant differences concern the negative control. Student’s *t*-test ** (*p* < 0.01), *** (*p* < 0.001).

For behavioral assays, a concentration of 0.25% H_2_O_2_ was selected, as this is the threshold at which the toxic effects of H_2_O_2_ begin to manifest while still allowing for enough adults to conduct the assays. The results indicated that H_2_O_2_ significantly increased motility in third-instar larvae after four hours of exposure (*p* < 0.001; Figure 5A) and in adult females previously exposed during the third-instar larval stage (*p* < 0.01; Figure 5B).

A summary of the statistical results from larval and adult behavioral assays is available in the Supplementary Materials (Table S2).

## 4. Discussion

An indicator commonly used to assess the teratogenic potential of a substance is the teratogenic index (TI); however, the literature does not clearly establish which developmental stages should be evaluated. In the present study, two scenarios were considered: (1) anomalies present only in living adults and (2) anomalies present in larvae, pupae, and adults. The EC_50_ for H_2_O_2,_ considering only adult anomalies, was 0.75%, whereas the EC_50_ including anomalies across all developmental stages was 0.36%. These concentrations were determined using graphical methods (Figures S1 and S2).

Based on these results, considering an LC_50_ of 0.16%, the TI was calculated as 0.21 when only adult anomalies were considered and 0.44 when anomalies in the larval, pupae, and adult stages were included. In both cases, the TI value was <3, classifying H_2_O_2_ as a compound of low teratogenic risk, according to the scale proposed by Jarques et al. (2020) [29]. This classification may correspond to FDA category C for human risk assessment, according to Lee et al. (2013) [21]. These results are consistent with previous reports for the *Xenopus laevis* and *Mus musculus* models [30].

It is important to note that higher TI values indicate greater teratogenic potential. This is particularly relevant because teratogenic effects observed at concentrations close to lethal levels may result from nonspecific toxicity rather than from targeted chemical interference with developmental pathways. Although TI threshold values can vary depending on experimental conditions, teratogenicity studies must consider the teratogenicity–lethality relationship to avoid over- or underestimation of a compound’s teratogenic potential [29]. Therefore, the TI value obtained when considering abnormalities across all developmental stages offers a more accurate representation of the teratogenic profile of hydrogen peroxide in the *Drosophila melanogaster* model.

The results demonstrated the teratogenic potential of hydrogen peroxide, evidenced by the morphological abnormalities observed across multiple developmental stages of *Drosophila melanogaster*. Nonetheless, the teratogenic index (TI) should be interpreted with caution, as several factors may influence its reduction. These factors include the exposure route, which may limit compound bioavailability and systemic absorption; the possibility that the larval stage does not constitute the critical period for toxicant exposure; and the presence of efficient cellular repair and detoxification mechanisms within the organism that mitigate developmental damage.

Regarding locomotion activity, larvae exhibited increased foraging behavior compared with the negative control and displayed more erratic movement patterns. In adults, flight activity increased, with higher levels recorded in females than in males. These findings are consistent with those reported by Grover et al. (2009) [23] for concentrations above and below 0.25%, and they support the hypothesis that behavioral alterations may be linked to the effects of hydrogen peroxide on circadian mechanisms, particularly the central circadian pacemaker.

Behavior in an insect such as *Drosophila melanogaster* is a complex trait influenced by genetic, environmental, and even social factors [31,32]. Specifically, regarding locomotor patterns, it has been demonstrated that, in natural populations, males exhibit higher locomotor activity, which facilitates mate searching and thus reproductive success, whereas females show reduced motility, as they prioritize foraging to secure the energetic resources necessary for egg production [33]. In the present study, an inverse pattern was observed in adults; however, the assay performed does not allow for the determination of the underlying causes of this behavioral shift. Based on the literature reports, these behavioral changes could be associated with metabolic alterations [34], sex-differential expression of genes encoding enzymes involved in neutralizing reactive oxygen species [23,35], neuronal death [36], premature aging and infertility [33,37], disruption of circadian rhythms [23], and even intralocus sexual conflict [33], among others. Therefore, further in-depth investigation is warranted.

Finally, it is important to acknowledge that the present study has certain limitations, including the absence of a positive control and the lack of mechanistic approaches to identify the molecular pathways underlying the observed effects. Nevertheless, these limitations highlight key areas for future research, particularly toward clarifying the mechanisms by which hydrogen peroxide and other oxidative stress-inducing environmental contaminants disrupt developmental processes at the cellular and organismal levels.

## 5. Conclusions

Hydrogen peroxide induces morphological abnormalities in *Drosophila melanogaster* beginning at concentrations of 0.75%, with the most severe effects occurring in the larval and pupal stages. Behavioral changes were detected at 0.25%, with greater impact observed in adult females. The results suggest that *Drosophila melanogaster* is a suitable model for investigating teratogenesis caused by xenobiotics that generate oxidative stress.

## Data Availability

The datasets used and analyzed during the current study are available from the corresponding author upon reasonable request.

## Supplementary Materials

The following supporting information can be downloaded at: https://www.mdpi.com/article/10.3390/biology14091122/s1. Figure S1: Median effective concentration (EC_50_) for H_2_O_2_ across all developmental stages; Figure S2: Median effective concentration (EC_50_) for H_2_O_2_ in viable adults; Table S1: Statistical summary of the percentage of morphological abnormalities induced by hydrogen peroxide in *Drosophila melanogaster*; Table S2: Statistical summary of the larval and adult behavioral assays.

## Abbreviations

The following abbreviations are used in this manuscript:

DNA	Desoxyribonucleic acid
EC_50_	Effective concentration 50%
FDA	Food and Drug Administration
LC_50_	Lethal Concentration 50%
ROS	Reactive oxygen species
SE	Standard error
TI	Teratogenic index
