# Supplementary material for: Morphological and Behavioral Abnormalities Induced by Hydrogen Peroxide in Drosophila melanogaster
Source: Biology (Basel). 2025 Aug 25;14(9):1122. doi: 10.3390/biology14091122 (PMC12467041; doi:10.3390/biology14091122)
Supplement: Supplementary file 1 [file biology-14-01122-s001.zip › biology-3783591-supplementary.pdf]

Supplementary Materials

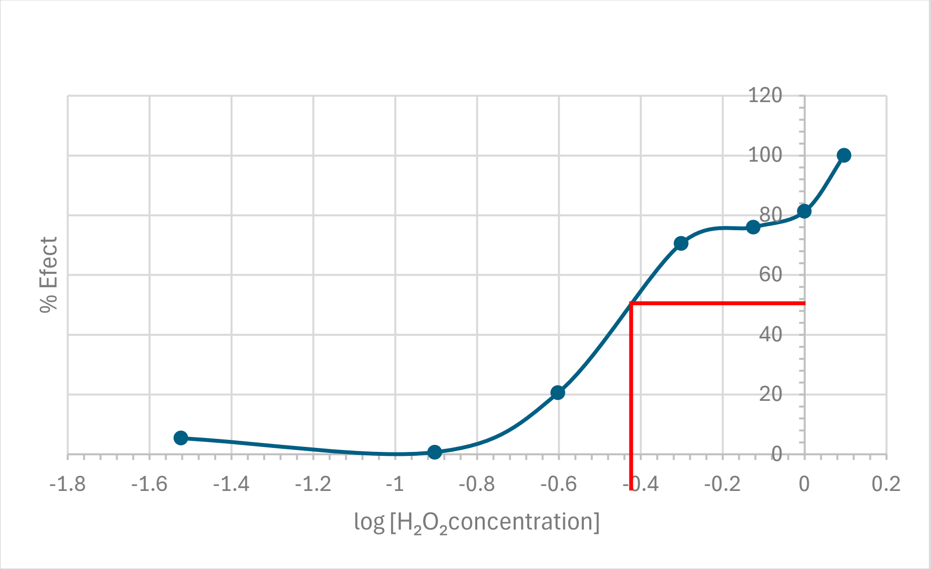

Figure S1. Effective concentration 50 (EC_{50}) considering the effect of H_2O_2 across all developmental stages.

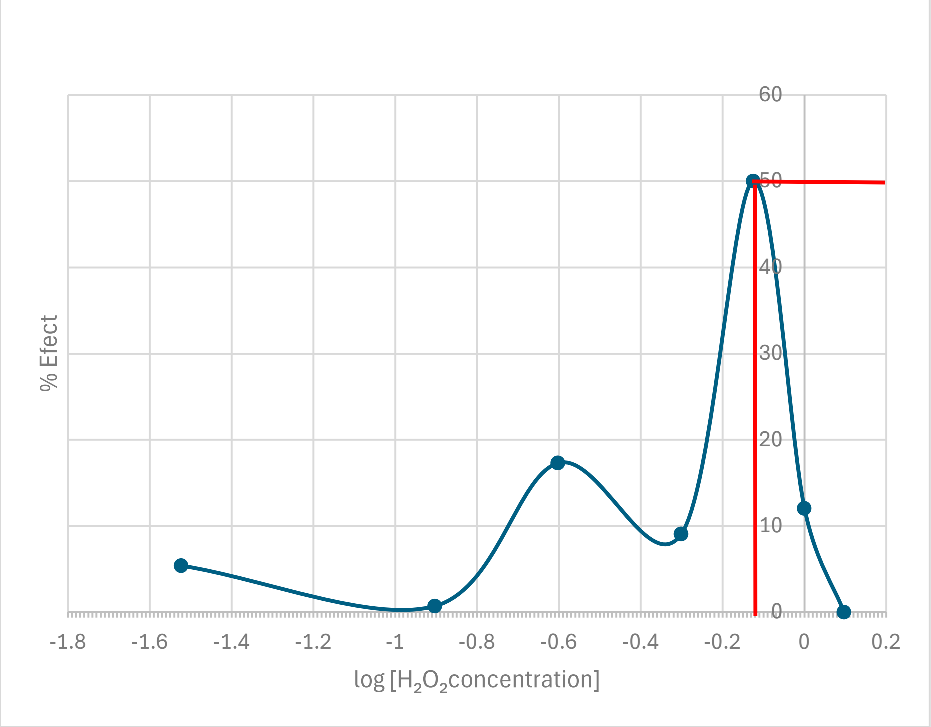

Figure S2: Effective concentration 50 (EC_{50}) considering the effect of H_2O_2 in viable adults.

Table S1. Statistical summary of the percentage of morphological abnormalities induced by hydrogen peroxide in *Drosophila melanogaster*

	% abnormalities in viable adults*	% abnormalities across all developmental stages**
**Experimental group**	Mean ± Standard Deviation	Mean ± Standard Deviation
**Negative control**	0.66± 1.15	0.66± 1.15
**0.03**	a	5.40±0
**0.125**	a	0.7±1.15
**0.25**	7.40±8.98	20.67±4.16
**0.5**	9.06±5.51	70.67±6.11
**0.75**	50±0	76±2.0
**1.0**	12.03±12.52	81.3±6.43
**1.25**	a	100±0

a, No morphological abnormalities were observed.
* It was calculated based on the % viability
** It was calculated based on n=50

Table S2. Statistical summary of the larval and adult behavioral assays

Experimental group	n/replicate	Mean ± Standard Deviation
	**Larval behavior assay**	
**Negative Control**	30	4.6± 4.12
**H_2O_2**	30	9.8± 2.43
	**Adult behavior assay**	
**Negative Control**	50	5.7± 1.33
**Females exposed to H_2O_2**	50	8.6± 1.0
**Negative Control**	50	6.6± 2.34
**Males exposed to H_2O_2**	50	5.8±1.0
